# Programmable synchronization enhanced MEMS resonant accelerometer

**DOI:** 10.1038/s41378-020-0170-2

**Published:** 2020-07-27

**Authors:** Liu Xu, Shudong Wang, Zhuangde Jiang, Xueyong Wei

**Affiliations:** State Key Laboratory for Manufacturing Systems Engineering, Xi’an Jiaotong University, Xi’an, 710049 China

**Keywords:** Electrical and electronic engineering, Sensors

## Abstract

Acceleration measurement is of great significance due to its extensive applications in military/industrial fields. In recent years, scientists have been pursuing methods to improve the performance of accelerometers, particularly through seeking new sensing mechanisms. Herein, we present a synchronized oscillator-based enhancement approach to realize a fivefold resolution improvement of a microelectromechanical resonant accelerometer. Through the unidirectional electrical coupling method, we achieved synchronization of the sensing oscillator of the microelectromechanical resonant accelerometer and an external reading oscillator, which remarkably enhanced the stability of the oscillation system to 19.4 ppb and the resolution of the accelerometer to 1.91 μg. In addition, the narrow synchronization bandwidth of conventional synchronized oscillators was discussed, and hence, we propose a novel frequency automatic tracking system to expand the synchronization bandwidth from 113 to 1246 Hz, which covers the full acceleration measurement range of ±1 g. For the first time, we utilized a unidirectional electrical synchronization mechanism to improve the resolution of resonant sensors. Our comprehensive scheme provides a general and powerful solution for performance enhancement of any microelectromechanical system (MEMS) resonant sensor, thereby enabling a wide spectrum of applications.

## Sensors: exploiting synchronization in developing high-resolution accelerometers

Researchers in China have first time exploited the natural phenomenon of synchronization into practical sensor design, which significantly enhanced the resolution of microelectromechanical systems (MEMS) accelerometers. A team led by X.W. and Z.J. at Xi’an Jiaotong University achieved synchronization of the sensing oscillator in the accelerometer and an external reading oscillator through the injection locking. The synchronization leads to a fivefold boost in the resolution of the accelerometer. The team also developed a frequency-tracking system to break through the limitation of the synchronization range thereby enabling the device to work in a wider dynamic range. In addition to the direct application in accelerometers, the technique can also be used to improve the performance of other MEMS resonant sensors.

## Introduction

High-precision microelectromechanical resonant accelerometers have attracted a great deal of interest due to their high sensitivity^1^, wide dynamic range^2^, and extensive potential applications in military/industrial fields, e.g., inertial navigation^3^, seismic detection^4^, and consumer electronics^5^. Through years of development, scientists have successfully solved many fundamental problems, such as topology structure optimization^6^, oscillation circuit noise suppression^7^, and long-term drift cancelation^8^. However, the nonlinearity of the MEMS resonator that emerges from the size effect becomes an important factor that affects the stability of the oscillator and the performance of the accelerometers^9^. The conventional solution is to minimize the nonlinearity of the resonators by reducing the excitation force or adjusting the balance of softening and hardening springs^10^, thus keeping the resonators from operating in a nonlinear range.

Counterintuitively, in recent years, nonlinear effects such as internal resonance^11^, mode localization^12^, mode matching^13^, and synchronization^14^ have been found to be beneficial for the performance of resonators, which can potentially be utilized to extend the detection limit of microelectromechanical resonant accelerometers. Spletzer et al.^15^ introduced the paradigm of mode-localized sensing and demonstrated that the relative changes of two coupled microcantilevers at the eigenstates due to external stress can be orders of greater than those at the resonance frequencies. Based on the same theory, Zhao et al.^16^ proposed a resolution-enhanced resonant accelerometer integrated with a stiffness tunable coupled microresonator system. Synchronization is another example of nonlinear behavior arising from the collective dynamics of coupled oscillators, which was recently observed in electrostatic coupled micro^17^ and nano^18^ resonators. The experimental results have demonstrated that the frequency stability of the synchronized oscillation system can be improved^19^ and the phase noise reduced^20^. In 2019, we proposed the first microelectromechanical resonant accelerometer based on synchronized double-ended tuning forks (DETFs), whose resolution was found to be improved by a factor of two^21^. However, as a brand-new sensing mechanism, the synchronization-based sensing scheme still faces many challenges, such as scale factor degradation, synchronizing bandwidth limitations, and coupling strength regulation.

In this paper, we propose a unidirectional electrical coupling method to achieve the synchronization of an MEMS resonant accelerometer and an external DETF resonator. In the first part, we describe the concept and the working regime of the synchronized oscillator resonant accelerometer. The frequency signal of the accelerometer is inputted to the external resonator as a synchronizing perturbation to establish a weak dynamic coupling oscillation system. In the following section, we describe static and dynamic calibration for the synchronized accelerometer, and the resolution improvement and excellent tracking performance of synchronization can be observed in the experimental results. Next, we establish the model and analyze why the synchronization bandwidth is not enough to meet the demand of the working range of the accelerometer at the level of existing resonator structural parameters. In light of this analysis, we propose a novel frequency automatic tracking system to dynamically expand the synchronization bandwidth of the synchronized oscillator resonant accelerometer. As a universal technique, our method can be utilized to optimize any kind of resonant sensor without compromising its original performance metrics, such as the measurement range, scale factor, or accuracy.

## Results and discussion

### The concept and design of a synchronized resonant accelerometer

Resonant mode decoupling has been historically regarded as a superior concept in the structural design of MEMS inertial devices^22^. Herein, we propose a mode decoupling method based on two separated MEMS resonators, i.e., a sensing resonator and a reading resonator. The sensing resonator is coupled to a mass so that it can sense the external acceleration through frequency shifts. A real-time monitoring system is utilized to let the sensing resonator and reading resonator oscillate in the synchronization state and to record the characteristic frequencies of the two. According to our previous study, an enhanced stability of the reading resonator can be found in the synchronization bandwidth^23^, so the accelerometer’s resolution can be boosted. The advantages of the proposed mode decoupling method are twofold. First, the optimization scheme is universal; i.e., for an MEMS resonant sensor of any kind, our scheme can provide a resolution improvement by simply utilizing an external reading resonator. Second, through isolating the sensing mode and the reading mode via two different resonators, we can realize a differential optimization of the two resonators. For instance, in the finite element method, we focus mainly on the scale factor and linearity of the sensing resonator, with additional focus on the *Q*-factor and nonlinearity of the reading resonator. Differential optimization allows for better performance starting at the beginning when the device is designed.

**Fig. 1:**
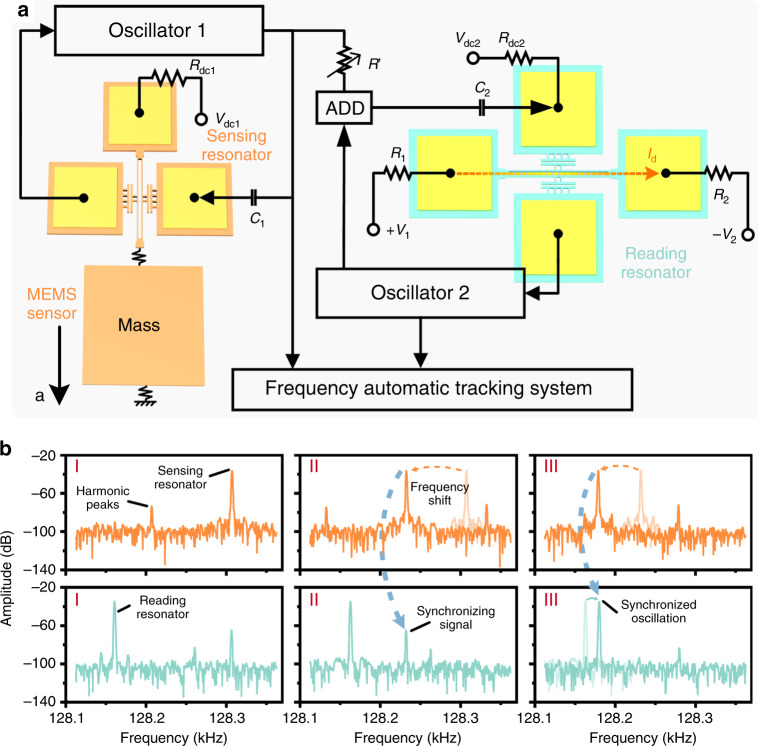
Working principle of the synchronized resonant accelerometer. **a** The proposed synchronized resonant accelerometer consists of a microelectromechanical resonant accelerometer, a reading resonator, and a frequency-tracking system. The concept behind the design is to decouple the sensing mode and the reading mode through two isolated resonators as well as to improve the resolution of the accelerometer. **b** Spectrum response of the sensing resonator (orange) and the reading resonator (cyan) during a typical synchronization process. Through the unidirectional electrical coupling method, a sidelobe can be observed in the spectrum of the reading resonator (stage I), which shifts as the induced external acceleration changes (stage II). When the sidelobe is close enough to the resonant peak of the reading resonator, the latter is synchronized to the former, and a jumping of the main peak can be observed (stage III).

As demonstrated in Fig. 1a, the programmable synchronizing oscillation-enhanced MEMS resonant accelerometer consists mainly of a microelectromechanical resonant accelerometer, a reading resonator and a frequency-tracking system. The sensing resonator and reading resonator are embedded in two self-excited oscillation circuits separately. The mass is subject to the variation in environmental acceleration that applies stress on the sensing resonator, thus tuning its frequency. Then, the frequency signal of the sensing resonator is transmitted to the reading resonator. According to the dynamic model of the synchronized oscillator, the remote electrical synchronizing signal applied to the reading resonator can be regarded as a perturbation, as this signal is much smaller than the excitation force of the reading resonator^23^. When the frequency of the perturbation is close enough to that of the reading resonator, the reading resonator will be synchronized to the perturbation, and accordingly, the frequency shift of the sensing resonator induced by the acceleration can be read out precisely from the reading resonator.

Figure 1b shows the spectrum response of the sensing resonator and the reading resonator during a typical synchronization process. At first, when the frequencies of the two are far from each other, they oscillate separately as two uncorrelated systems (stage I). When the sensing resonator is tuned by the external acceleration, the shift of the resonant peak can be observed, and the synchronizing signal approaches the main spectrum peak of the reading resonator (stage II). Eventually, when both frequencies are close enough, the sidelobe and the main peak of the reading resonator blend together, and synchronization happens (stage III). As there is no feedback signal from the reading resonator to the sensing resonator, the oscillation state of the sensing resonator is not influenced by the reading resonator, which indicates that such a unidirectional electrical coupling method does not affect the output precision of the sensor^20^.

### Performance calibration of a synchronized resonant accelerometer

**Fig. 2:**
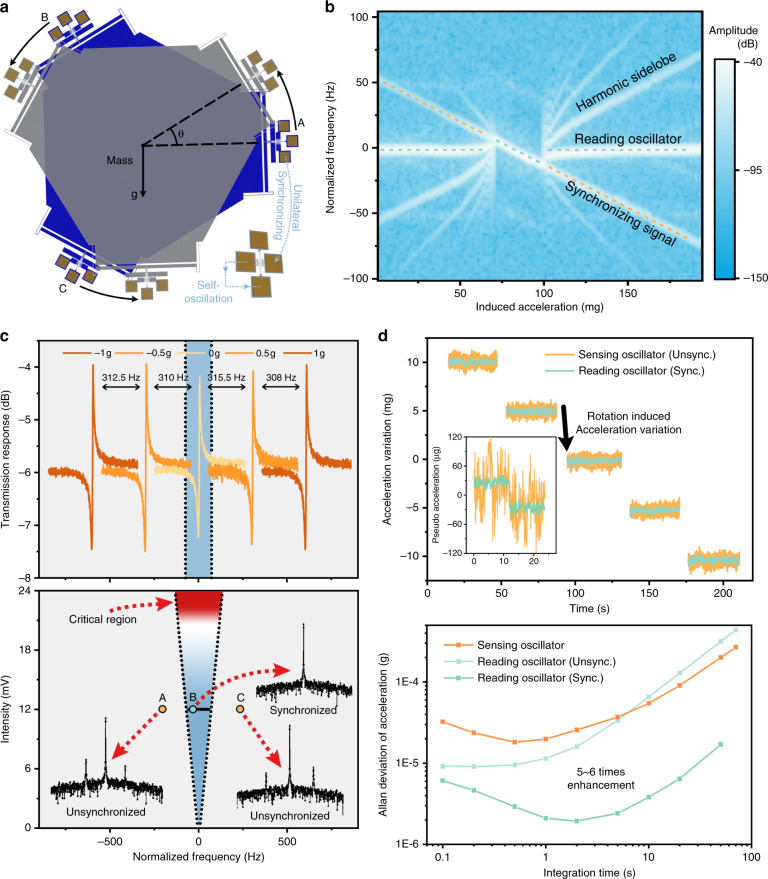
Static performance of the system. **a** During the experiment, the resonant accelerometer was installed on a rotary table to sense the acceleration change, while the reading resonator was statically arranged. The sensing resonator and the reading resonator were synchronized through the unidirectional electrical coupling method. **b** The spectrum response of the reading resonator when the external acceleration changed. Each vertical slice in the figure represents a 10 s average of the spectrum response. An obvious synchronization range can be observed in the middle of the graph, where all other sidelobes vanish. When entering and leaving the synchronization range, the system plunges into chaos, and all the resonant peaks are very disordered. **c** The synchronization range determines the measurement range of the synchronized resonant accelerometer, which is linearly dependent on the perturbation intensity. The blue area in the graph indicates the proportion of the synchronization range to the whole measurement range of ±1 g. Increasing the voltage of the synchronizing signal is an effective way to expand the synchronization range; however, an overlarge perturbation intensity (red region) might destroy the oscillation rhythm of the reading resonator and even threaten its integrity. When the system is out of the synchronization range (points A and C), the synchronized resonant accelerometer operates as two independent oscillators. **d** Resolution test of the synchronized resonant accelerometer. As the external acceleration changes, the reading oscillator shifts stepwise with the sensing oscillator. According to the calculation of the Allan deviation, the stability of the reading oscillator after synchronization was enhanced from 90.8 to 19.4 ppb, which was an improvement of 5–6 times, while that of the sensing oscillator remained 181.4 ppb. The calculated resolution of the original resonant accelerometer is ~17.3 μg. However, with the help of the synchronization enhancement, the resolution of the synchronized resonant accelerometer is increased ninefold to 1.91 μg.

In our proposed synchronized resonant accelerometer, the sensing resonator and reading resonator have different functions: the sensing resonator can ‘actively’ change its frequency in responding to the external acceleration, and the external reading resonator ‘passively’ synchronizes with the sensing resonator and outputs its dynamic response. Our proposed microelectromechanical resonant accelerometer consists of a hexagon mass block, six force amplifiers, and three sensing resonators, as shown in Fig. 2a. The mass is placed in the center, with the sensing resonators and force amplifiers radially arrayed around it. The proof mass is subject to environmental acceleration and transmits the inertial force to the end of the sensing resonators through the force amplifiers, thus changing the resonant frequencies of the sensing resonators ${\Delta f = f_0\left( {0.1475\frac{{TL^2}}{{12EI}}} \right)}$, where *T* is the axial force, *I* is the rotational inertia, *E* is Young’s modulus of silicon, and *L* is the length of the sensing resonator.

In the experiment, the resonant accelerometer was vertically installed on a rotatory table with a positional accuracy of 0.01° and then placed in a vacuum chamber to ensure a low damping ratio. The quality factor of the sensing resonator was ~12,000 in a vacuum chamber at pressures below 2 Pa at room temperature. The sensing resonator and reading resonator were embedded in two self-oscillation circuits separately, and the resonant frequency was recorded in real time. Figure 2b presents the frequency spectrum variation of the reading oscillator during the rotation test. As the rotation angle varied, the corresponding acceleration changed from 0 g to 200 mg, and the frequency of the synchronizing signal originating from the active sensing oscillator changed linearly. However, the frequency of the reading oscillator (*f*_RO_) was independent of the tilting angle until synchronization occurred. In the synchronization range, the reading oscillator tightly tracked the changes in the sensing oscillator, while the ‘sidelobes’ also vanished. When *f*_*RO*_ moved out of the synchronization range, the synchronization state broke down, and accordingly, the reading oscillator and sensing oscillator ran independently.

Figure 2c demonstrates the synchronization bandwidth (blue) over the full range of ±1 g (gray). For sensing resonator A of our accelerometer with a scale factor of 623 Hz/g, the typical synchronization range (113 Hz) covers only 9.07%, which means that the effective working range of the synchronized resonant accelerometer might shrink. Fortunately, the synchronization range can be tuned by varying the perturbation strength according to the following formula (see Supplementary section 1):

1 $${\mathrm{Sync}}\,.\,{\mathrm{Bandwidth}} = 2\left| {\Delta {\mathit{\Omega}} } \right| = \frac{{8{{{E}}}}}{{\pi {Q}s_0}}\left[ {\left( {\frac{{3{Q}\beta a_0^2}}{{2\omega _0}}} \right)^2 + 1} \right]^{1/2}$$

where Δ*Ω* is the frequency mismatch between the sensing oscillator and the reading oscillator, *E* is the amplitude of the perturbation signal from the sensing oscillator, *Q* is the quality factor of the reading resonator, *s*_0_ is the amplitude of the feedback excitation voltage of the reading resonator, *a*_0_ is the vibration amplitude, *ω*_0_ is its characteristic frequency, and *β* is the nonlinearity.

According to the numerical simulation, it can be found that the synchronization bandwidth is proportional to the intensity of the perturbation signal, which coincides with the experimental results shown in Fig. 2c. However, in practical situations, when *E* exceeds 20 mV, the synchronization state enters a critical region (red), as the overlarge perturbation intensity could destroy the vibration rhythm of the MEMS oscillator and even threaten the integrity of the resonator.

The performance of our proposed synchronized resonant accelerometer within the synchronization range was further determined through an acceleration resolution experiment, as demonstrated in Fig. 2d. When the rotary table rotated with a step of 0.286°, the acceleration applied on the MEMS accelerometer was shifted ~5 mg for each step. The frequency of the sensing resonator was tuned according to the applied acceleration, while that of the reading resonator closely followed, and significant noise suppression was observed. This enhancement was even more obvious when the induced acceleration variation was smaller. As demonstrated in the inserted plot of Fig. 2d, we changed the direct current excitation voltage $\left( {\Delta V = 0.01\,\mathrm{V}} \right)$ of the sensing resonator to simulate a tiny acceleration with a sudden change and then recorded the frequency change of the sensing oscillator and that of the reading oscillator in synchronization. In a comparison with the frequency data of the sensing oscillator (orange), it was easy to distinguish the pseudo acceleration of ~60 μg from the reading oscillator (cyan) without any output delay. The excellent tracking performance of the reading oscillator reveals that such a synchronizing oscillation enhancement method is qualified for accelerometer measurement.

To demonstrate the frequency stability improvement and phase noise suppression of such a method, we characterized the frequency-associated Allan deviations of the sensing oscillator, reading oscillator with and without synchronization under the resting condition. It is clear that the short-term frequency stability of the sensing oscillator and reading oscillator before synchronization are close to each other, while that of the reading oscillator is boosted 5–6 times to 19.4 ppb after synchronization. It is worth noting that the frequency fluctuation is minimized after synchronization, and to reach the minimum deviation, it takes ~2 s longer than with the free running oscillator. The reading oscillator under the synchronization state has a suppressed noise floor of ~1 μg/$\sqrt {\text{Hz}}$ in the frequency range from 1 to 5 Hz, while this floor is ~5.31 μg/$\sqrt {\text{Hz}}$ under the nonsynchronization state (see Supplementary section 3). This proves that synchronization has a significant noise suppression effect. The resolution *R* of our accelerometer is calculated by the formula $R = \frac{{A \cdot f_0}}{S}$, where *A* is the minimum Allan deviation of the reading oscillator, *f*_0_ is the characteristic frequency of the reading oscillator, and *S* is the scale factor of the resonant accelerometer. Therefore, the resolution of our synchronized resonant accelerometer is ninefold increased to 1.91 μg as compared with 17.3 μg of the original resonant accelerometer without synchronization.

**Fig. 3:**
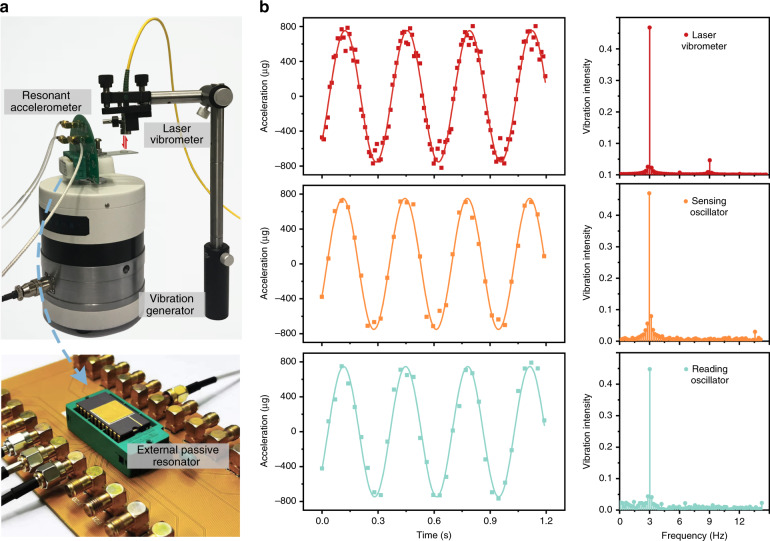
Dynamic performance of the system. **a** The experimental setup of the dynamic test. The resonant accelerometer was placed on a shaker to sense the dynamic acceleration. The external reading oscillator was synchronized with the sensing oscillator and closely tracked its response with a sampling rate of 50 ms. During the vibration, a laser vibrometer was utilized to provide a standard calibration. **b** The experimental results of the dynamic test. Under the low-frequency vibration of 3 Hz, the amplitudes of the sensing oscillator and reading oscillator were both nearly equal to that of the laser vibrometer. These results meant that the reading oscillator could perfectly track and read out the dynamic environmental acceleration.

The dynamic response of our proposed synchronized resonant accelerometer was investigated through a vibrating calibration system, as demonstrated in Fig. 3a. The resonant accelerometer was placed on the vibration table to sense low-frequency dynamic acceleration signals, while the reading resonator was statically placed and synchronized with the sensing resonator. A laser vibrometer was utilized as a reference to the vibrating acceleration. Figure 3b shows the real-time output of the sensing oscillator and reading oscillator when a sinusoidal vibration signal was generated by the vibration table with a low frequency of 3 Hz. The measured average peak-to-peak amplitudes of the sensing oscillator and reading oscillator were 750.6 and 757.5 μg, respectively, which were nearly equal to the experimental amplitude of the laser vibrometer of 752.1 μg. It is worth noting that the phase response of the sensing oscillator and reading oscillator was exactly matched with the vibrometer, which means that the reading oscillator could perfectly track and read out the dynamic environmental acceleration. The results of fast Fourier transformation further confirm that such a system can achieve a reliable amplitude and frequency measurement of the dynamic vibration without any accuracy loss.

### Synchronized resonant accelerometer with an expended measurement range

Although the discussed synchronized resonant accelerometer shows good feasibility and reliability, its inherent narrow synchronization bandwidth limits its measurement range. If the acceleration-induced frequency shift is beyond the synchronization range, the synchronization state will break up, and the proposed accelerometer system will operate as two discrete units, i.e., a resonant accelerometer and a reading oscillator. Therefore, the sensing oscillator and the reading oscillator have to remain synchronized within the desired working range. For two synchronous self-oscillators, the synchronization range can be described by:

2 $$H = \frac{{8{E}}}{{\pi nQs_0}}\left[ {\left( {\frac{{3Q\beta a_0^2}}{{2\omega _0}}} \right)^2 + 1} \right]^{1/2} - \omega _0 \cdot P \ge 0$$

where $\omega _0 \cdot P$ is the target working range. The maximum value of the optimal solution (*H*) can be obtained by the Lagrange multiplier.

**Fig. 4:**
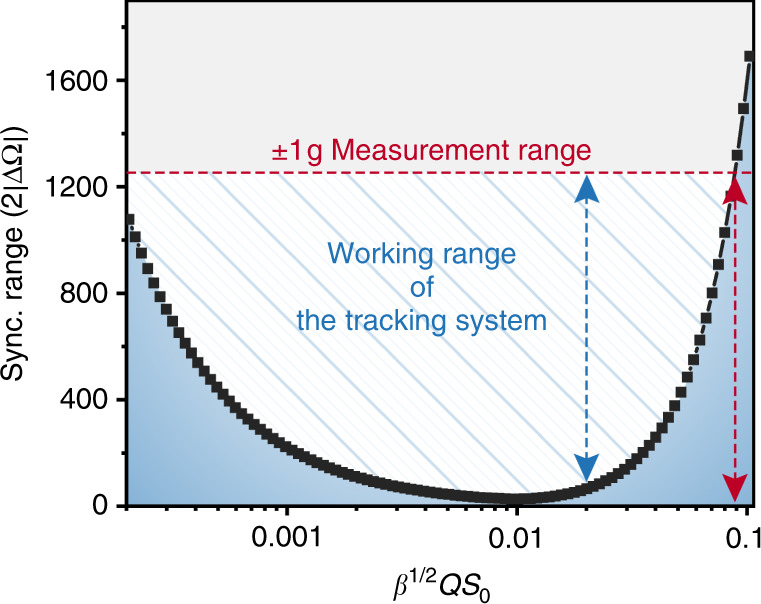
The prediction of the synchronization bandwidth. Compared with the target working range of ±1 g, the current reading oscillator has a rather small synchronization bandwidth, which could not readily cover the range required by the accelerometer. Although it was theoretically possible to maximize the synchronization bandwidth by optimizing the structural parameter of the resonator, $\beta ^{1/2}Qs_0\sim 0.09$ implies that the quality factor *Q* of the resonator must reach at least 100k while the structure still has relatively strong nonlinearity. Moreover, a strong *s*_0_ might pose a threat to the integrity of the MEMS devices. Therefore, a frequency automatic tracking system is needed to widen the working range of the synchronized resonant accelerometer.

Figure 4 demonstrates the functional relationship between the synchronization range and $\beta ^{1/2}Qs_0$. When $\beta ^{1/2}Qs_0$ increases from 10^−4^ to 10^−1^, the synchronization range first decreases and then increases. According to our measured structural parameters of the reading resonator $\left( {\beta ^{1/2}Qs_0 = 0.02} \right)$, the synchronization bandwidth of the synchronized resonant accelerometer is only 113 Hz, which is far less than the ±1 g working range of 1246 Hz (blue line). According to the theoretical prediction, the synchronization range cannot cover the required working range of ±1 g until $\beta ^{1/2}Qs_0$ is greater than 0.09 (red line), which indicates that the quality factor of the reading resonator needs to reach at least 100k while still having a strong nonlinearity. However, this is a huge challenge at the level of design, fabrication, and packaging. At present, the reported synchronization range accounts for less than 3‰ of the characteristic frequency^18,23–25^, depending on the structural parameters of the resonator. Therefore, to ensure a certain working range of the synchronized resonant accelerometer, it was necessary to adopt new techniques to cover the working range by the synchronization range.

**Fig. 5:**
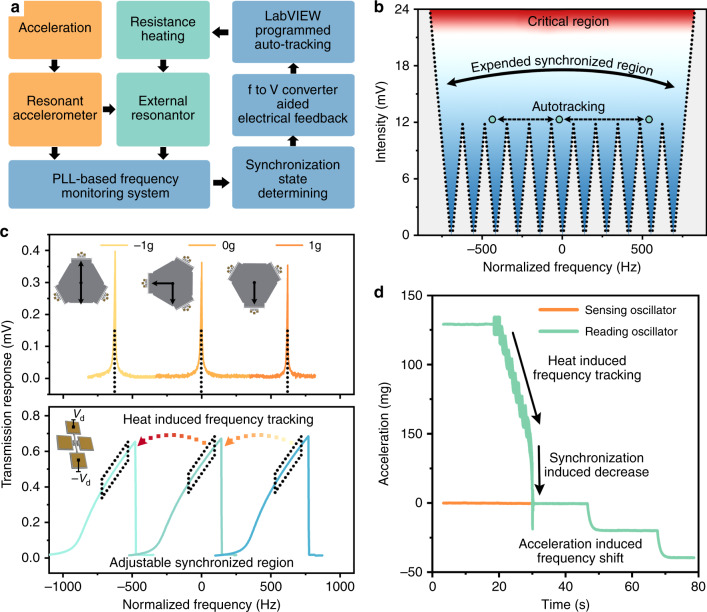
Working principle of the frequency automatic tracking system. **a** Systematic diagram of the frequency automatic tracking system. When the frequency difference exceeds the preset frequency threshold, the synchronization determining module can provide algorithmic feedback to the frequency spillage, thus adjusting the frequency of the reading oscillator through the Joule heating effect. The whole automatic tracking process runs in a programmable control environment. **b** The center frequency of the synchronization range can gradually shift and ultimately completely cover the working range required. **c** The open-loop frequency response of the sensing resonator and reading resonator under the monitoring of the tracking system. The nonlinear frequency response of the reading resonator is shifted under Joule heating, which perfectly tracks the changes in the sensing resonator. **d** Demonstration experiment of the tracking system. When the frequencies of the sensing oscillator and reading oscillator are far from each other, the tracking system can sense the difference and then change the reading oscillator until the two are synchronized.

In this way, we considered adjusting the frequency of the reading resonator through Joule heating, thus dynamically breaking through the limitation of the synchronization range. Herein, we propose a frequency automatic tracking system to achieve a synchronization range with adjustable central frequencies. Figure 5a shows the structure diagram of the tracking system, which consists mainly of the hardware module, i.e., the resonant accelerometer, the external reading resonator, the frequency monitoring system, and the software module, i.e., the synchronization state determining method, the electrical feedback, and the proportion integration differentiation (PID) control. When the frequency of the sensing resonator changes significantly due to the external acceleration and exceeds the synchronization range, the monitoring system recognizes the change and determines whether the synchronization state is broken. If so, our system can automatically calculate the frequency difference between the sensing resonator and reading resonator and then further estimate how much feedback voltage is needed. This feedback voltage is applied on both ends of the reading resonator to increase or decrease its temperature. In this way, the frequency difference is reduced, and hence, synchronization is restored.

Figure 5b shows the theoretical effectiveness of our proposed frequency automatic tracking system. When the unidirectional synchronizing voltage is 12 mV, the numerical simulation shows that the synchronization bandwidth is only 113 Hz. When the frequency shift caused by the environmental acceleration variation exceeds the synchronization range, the PID control module applies a certain drain current *I*_d_ on the reading resonator. According to the Joule heating effect, the characteristic frequency of the reading resonator changes, which corresponds to a shift of the synchronization range in the spectrum (see Supplementary section 2). Therefore, the adjustable synchronization range can theoretically cover the full range (the blue area). It is worth noting that the reading resonator is installed far from the sensing resonator to prevent its thermal effect from affecting the output precision of the sensor.

Figure 5c demonstrates a typical working procedure of the frequency automatic tracking system during the open-loop test. In the experiment, the in-plane rotation of the resonant accelerometer causes an acceleration variation from 1 to −1 g, and the frequency of the sensing resonator decreases by ~1246 Hz. When the frequency difference Δ*f* exceeds the preset frequency threshold, the frequency-to-voltage conversion algorithm in the PID control module (see Supplementary section 2) can accurately calculate the needed drain current *I*_d_ and then tune the resonance frequency through the induced temperature. The purpose of PID control is to adjust the drain current of the resonator and determine the optimal parameters to minimize the control time. Therefore, taking the frequency difference Δ*f* as the input of the system and voltage as the output, we can obtain the classical second-order transfer function by:

3 $$\Phi \left( s \right) = \frac{K}{{Ts^2 + s + K}}e^{ - \tau _0s} = \frac{{\omega _n^2}}{{s^2 + 2\zeta \omega _ns + \omega _n^2}}e^{ - \tau _0s}$$

where $\Phi (s)$ is the transfer function, *T* is the time constant, *s* is the Laplace transform variable, *K* is the gain coefficient, $\omega _n = \sqrt {K/T}$ is the natural frequency, *ξ* is the damping, $e^{ - \tau _0s}$ is the delay element, and *τ*_0_ is the delay time. The PID control method can gradually make the reading resonator track the variation of the sensing resonator until they ultimately achieve synchronization again. In the transfer function, the response time *T* is determined by the gain coefficient *K* and delay time *τ*; it also depends on the compatibility of the resonator with the system. In the open-loop test, the sensing resonator operated in its linear region, while the reading resonator operated in its nonlinear region, as shown in Fig. 5c. This is the result of comprehensive optimization for the accuracy of the sensor output, the synchronization bandwidth and the frequency stability of the oscillator. Figure 5d shows the experimental results of the sensing oscillator and reading oscillator under the control of the tracking system. In the experiment, the sensing oscillator and reading oscillator were embedded in two separated self-oscillation circuits, and the dual channels of the frequency counter were used to simultaneously acquire the frequency of sensing oscillator *f*_SO_ and the frequency of reading oscillator *f*_RO_. These frequency data were fed into a LabVIEW program to implement the PID control process. At first, when the frequencies of the sensing oscillator (brown line) and the reading oscillator (green line) were separated, the system could recognize that the Δ*f* was greater than the preset frequency threshold *f*_th_ and thus that frequency compensation was required. Under PID control, *f*_RO_ approached the *f*_SO_ step by step at a speed of 7 Hz/s. When they were close enough, *f*_RO_ suddenly dropped to match *f*_SO_, which means the synchronization state was reconstructed (see Supplementary Fig. S3). Subsequently, when the resonant accelerometer was applied to an external acceleration, *f*_SO_ and *f*_RO_ changed equally at the same time.

## Conclusion

Sensing mechanisms based on multiresonators have attracted increasing interest in recent years, e.g., for mode localization, mode coupling, and synchronization. The coupling strength among the resonator arrays is critical to the sensitivity and resolution. However, for most of the proposed sensors, the strength can be difficult to control. In addition, the interaction force between the sensing resonator and the reading resonator may damage the scale factor of the sensor. Therefore, we propose a unidirectional electrical synchronization method that has a larger tunability and does not affect the scale factor of the sensing resonator. In addition, by isolating the sensing mode and the reading mode through two different resonators, we can realize a differential optimization of the two resonators, thus achieving better performance. The resolution of the proposed synchronized resonant accelerometer is improved to 1.91 μg. The noise floor of the accelerometer is suppressed from 5.3 to 1 μg/$\sqrt {\text{Hz}}$ and the accelerometer random walk is improved to 2.05 μg/$\sqrt s$. Based on its native synchronization range, our synchronized resonant accelerometer is suitable for the measurement of low-frequency acceleration with a low amplitude. When the acceleration-induced frequency shift is beyond the synchronization range, the proposed accelerometer system first operates as two discrete units, i.e., a resonant accelerometer and a reading oscillator. Then, with the help of the frequency automatic tracking system, the reading oscillator can track the frequency of the sensing oscillator and become synchronized, thus further improving the stability of the resonant accelerometer. In this way, the tracking system can expand the synchronization bandwidth from 113 to 1246 Hz, which covers the full acceleration measurement range of ±1 g. Therefore, the proposed method can be utilized to improve the resolution of any resonant sensor without compromising its original performance parameters, such as the measurement range, scale factor, or accuracy.

## Supplementary information

Supplementary Information

Supplementary Video
