# Supplementary material for: Programmable synchronization enhanced MEMS resonant accelerometer
Source: Microsyst Nanoeng. 2020 Jul 27;6:63. doi: 10.1038/s41378-020-0170-2 (PMC8433445; doi:10.1038/s41378-020-0170-2)
Supplement: Supplementary file 1 — Supplementary Information [file 41378_2020_170_MOESM1_ESM.docx]

**Supplementary Information**

**Supplementary Section 1. Deviation of synchronization range and working range for synchronized resonant accelerometer.**

To precisely predict the synchronization range with the nonlinear Duffing term, we describe the normal displacement equation based on Newton’ equation by:

$m\ddot{z}+\gamma\dot{z}+kz+k_{3}z^{3}=S_{0}\cos\left( \emptyset+\emptyset_{0} \right)+\Xi f_{s}(t)$ (S1)

Where z(t) is the displacement from equilibrium, *m* is the mass of synchronized DETF, $\gamma$ is the damping parameter, *k* is the elastic stiffness, $k_{3}$ is the cubic nonlinear stiffness, $S_{0}$ is the feedback force amplitude and $\Xi$ is external perturbation amplitude, $\emptyset_{0}$ is the feedback phase ($\emptyset_{0}=\frac{\pi}{2}$), $f_{s}(t)$ is the square wave function. In this case, $f_{s}(t)$ as the external perturbation signal to inject into the MEMS oscillator, which is regarded as external force in nonlinear Duffing equation.

Normalizing Eq. S1 with the total mass *m* yields,

$\ddot{z}+Q^{-1}z+z+\beta z^{3}=s_{0}\cos\left( \phi+\phi_{0} \right)+Ef_{s}(t)$ (S2)

Where *Q* is the quality factor and $Q=\frac{\omega_{0}}{\gamma}$, $\beta=\frac{k_{3}}{k}$, $s_{0}=\frac{S_{0}}{k}$ and $E=\frac{\Xi}{k}$*.*

The square wave can be regarded as the superposition of infinite trigonometric signals, the higher order harmonic signal can de decomposed by Fast Fourier Transform (FFT). The square wave function $f_{s}(t)$ can be given by:

$f_{s}\left( t \right)=sgn\left( cos\phi_{t} \right)=\frac{4}{\pi}\sum_{n=1,3,5\cdots}^{\infty} \frac{1}{n}sinn\omega_{s}t$ (S3)

Assuming the solution of the form:

$z\left( t \right)=a_{0}cos\emptyset=a_{0}cos\omega_{0}t$ (S4)

Without perturbation signal ($f_{s}=0$), we can obtain the expression of frequency $\omega_{0}$ and amplitude $a_{0}$ by using harmonic balance method:

$a_{0}=\frac{Q\cdot s_{0}}{\omega_{0}}$ (S5)

$\omega_{0}=\frac{1}{\sqrt{2}}{[1+{(1+3\beta Q^{2}s_{0}^{2})}^{1/2}]}^{1/2}$ (S6)

In the case of high order synchronization, the *N* order harmonic of the square wave as the perturbation signal interact with MEMS oscillator ($\Delta\omega=\omega_{0}-n\omega_{s}$), and others harmonic can be ignored. Therefore, the square wave can be simply expressed as:

$f_{s}(t)\approx\frac{4}{\pi}\frac{1}{n}sinn\omega_{s}t$ (S7)

Under the perturbation signal ($f_{s}\neq0$), we can get the expression of synchronization range $\left[ \omega_{0}-\Delta\Omega,\omega_{0}+\Delta\Omega\right]$ as follow:

$Sync. Range=2\left| \Delta\Omega\right|=\frac{8E}{\pi nQs_{0}}\left[ \left( \frac{3Q\beta a_{0}^{2}}{2\omega_{0}} \right)^{2}+1 \right]^{1/2}$ (S8)

For the Eq. S8, we can see that the synchronization range are simply proportional to the quality factor, the perturbation intensity $\frac{E}{s_{0}}$ and Duffing nonlinear $\beta a_{0}^{2}$. In order to ensure the certain working range of MEMS accelerometer, it is necessary to cover the working range by synchronization range. So it satisfies that:

$\boldsymbol{H}=\frac{8E}{\pi nQs_{0}}\left[ \left( \frac{3Q\beta a_{0}^{2}}{2\omega_{0}} \right)^{2}+1 \right]^{1/2}-\omega_{0}\cdot P\geq0$ (S9)

The maximum value of the optimal solution (***H***) can be obtained by the Lagrange Multiplier. In this paper, the frequency of sensing oscillator and reading oscillator are approximate each other, so the synchronization order is set by one ($n=1$).

**Supplementary Section 2. The detail of frequency automatic tracking systems.**

**Ⅰ. The effect of Joule heating in microresonator.**

In order to better explain the implementation mechanism of frequency tuning, we simulated the Joule heating effect using electric currents and heat transfer options in COMSOL. The simulation results show that when the resonator is heated by the drain current $I_{d}$, high temperature mainly distributed at the center part of the beam and temperature gradually decreases from the center part to anchor part as shown in figure S1 (a). The larger the drain current, the more heat generated by the resistor and the higher the body temperature. The detailed temperature distribution results are shown in figure S1 (b). Increasing temperature will change the Yong’s modulus of the resonator, eventually leading to shift of the resonant frequency, which can be also called by TCF (temperature coefficient of frequency). The relationship between resonant frequency and drain current can be expressed by:

$f_{TCF}=\frac{\beta^{2}\sqrt{E_{0}I}}{2\pi l^{2}}\cdot\sqrt{\frac{8k_{th}A^{2}+c_{\eta}I_{d}^{2}}{\rho A}}, where c_{\eta}=\rho_{e}\cdot TCE\cdot l^{2}$ (S10)

Where, $\beta$ is the mode constant, $E_{0}$ is Yong’s modules at room temperature, $I$ is the moment, $l$ is the length of beam, $k_{th}$ is the thermal conductivity, $\rho$ is the density, $A$ is the cross-sectional area, $\rho_{e}$ is the electrical resistivity. For silicon, the change in Yong’s modulus with temperature, the TCE, is approximately -63.7 ppm/℃ at normal operating temperatures.

The relationship between drain current $I_{d}$ and the resonant frequency in the experiment is shown in figure S1 (c). Within a small range of drain current $I_{d}$, there is a linear negative relationship between the two. This is fully consistent with the theoretical results.

The inhomogeneous distribution of thermal energy on the resonator body will soften the stiffness. The relationship between drain current $I_{d}$ and resonator body’s temperature $T_{0}$ can be described by:

$T_{0}=\frac{I_{d}^{2}\rho_{e}L^{2}}{8w_{b}h^{2}\kappa_{th}}+T_{b}$ (S11)

Where $\rho_{e}$ is the density, $\kappa_{th}$ is the thermal diffusivity, *h* is the beam thickness, *L* is the length, $w_{b}$ is the width, $T_{b}$ is the ambient temperature. Based on the temperature coefficient of frequency (TCF), the relationship between compensation voltage and external frequency can be described by:

$\Delta V=\left| V_{1}-V_{2} \right|=\frac{(R_{0}+R_{1}+R_{2})\cdot(-b+\sqrt{b^{2}-4a(c-Nf_{0})})}{2a}$ (S12)

Where, $\Delta V$ is the voltage difference of double ended of DETF, $V_{1}$ is the voltage of first DC voltage source, $V_{2}$ is the voltage of second DC voltage source, $R_{0}$ is the resistance of resonator body, $R_{1}$, $R_{2}$ are the first and second divider resistor, *a* is the third thermo-frequency factor, *b* is the second thermo-frequency factor, *c* is the first thermo-frequency factor, *N* is the synchronization order.


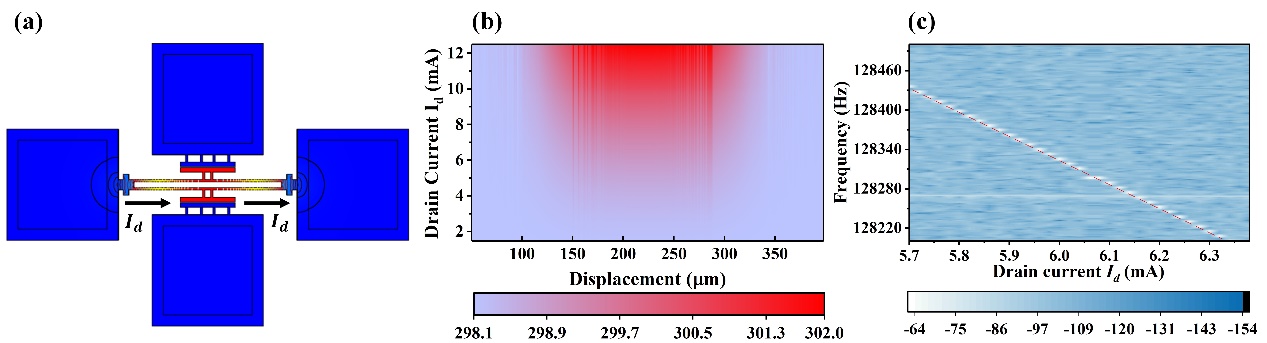


**Supplementary Figure S1.** The effect of Joule heating in microresonator. (a) Simulation of temperature distribution of resonator due to Joule heating effect. (b) Simulation results of temperature contour map with various drain current $I_{d}$ on the resonant structure. (c) Experimental results of spectrum response of frequency shift with various drain current $I_{d}$.

**Ⅱ. The algorithm flow chart of tracking system.**


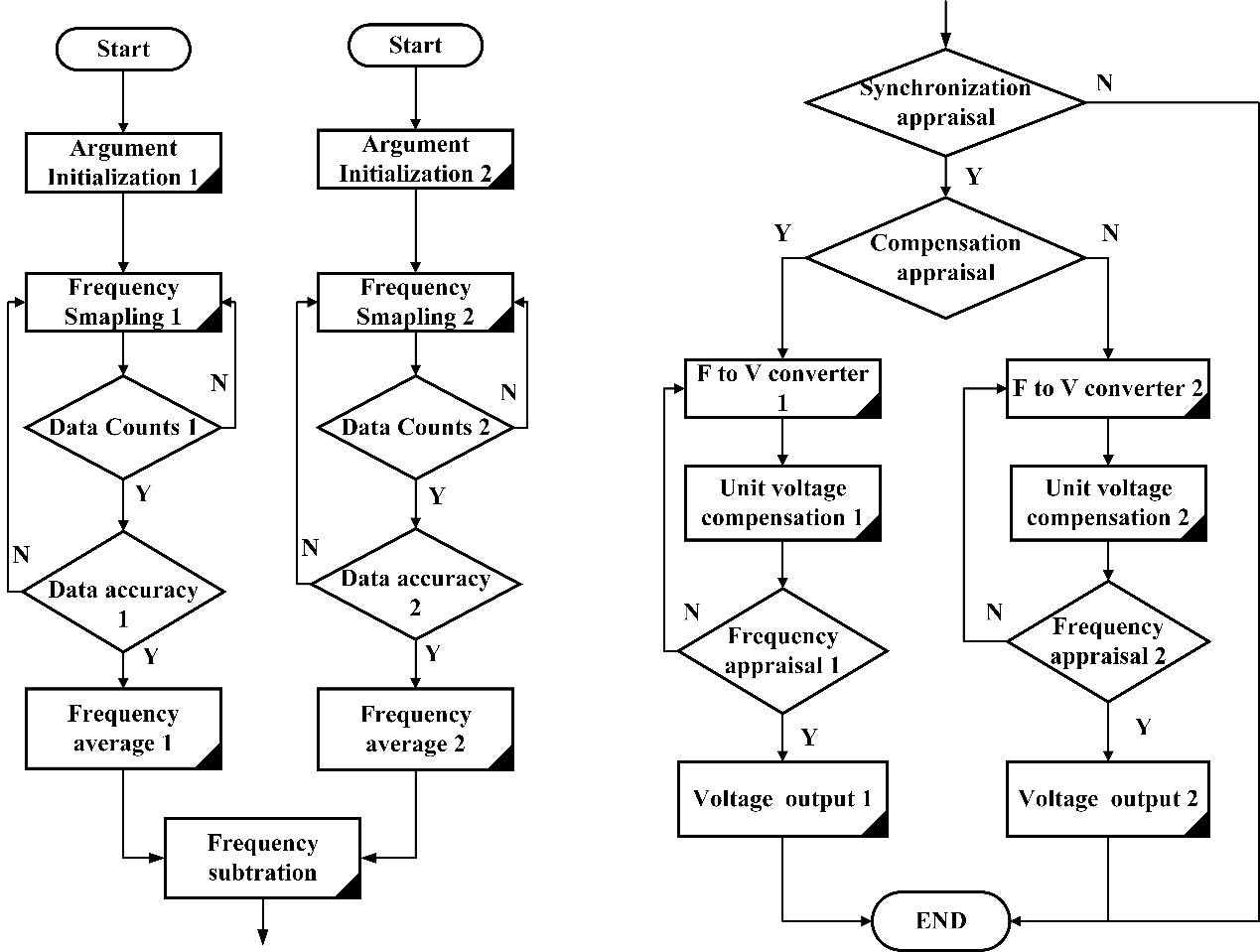


**Supplementary Figure S2.** The flow chart of automatic frequency tracking system. The working process of programmable frequency-tracking system is as follows: the frequency counter periodically readout the output signal of the sensing and reading oscillators and transmits the frequency data to computer. After filtering and averaging the frequency data, the system will judge whether the two oscillators are in synchronization state based on the synchronization appraisal. If under the non-synchronization state, the system will choose to perform either positive frequency compensation or negative frequency compensation according to the compensation appraisal. The value of frequency compensation will be converted into the corresponding voltage value by Eq. (12) and applied to the reading resonator.

**Ⅲ. The experimental results of PID control**


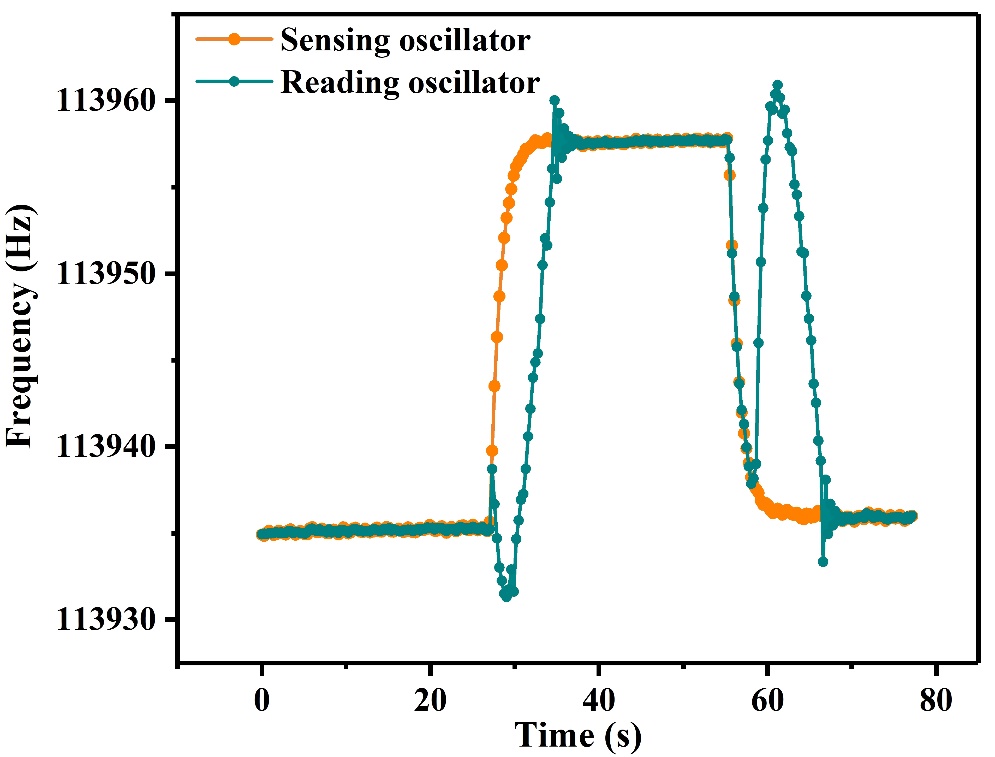


**Supplementary Figure S3.** The experimental results of frequency automatic tracking system. In order to better present the complete process of positive and negative frequency compensation, we changed the parameters of PID control. When the frequency shifts of sensing oscillator exceed frequency threshold $f_{th}$, the system could recognize that frequency compensation was required. Within 27s~40s, it belongs to the process of positive frequency compensation. The frequency of reading oscillator forward approached the frequency of sensing oscillator under the PID control. Within 58s~70s, it belongs to the process of negative frequency compensation, which is completely reverse to the positive compensation. It is worth noting that the speed of negative frequency compensation ($\nu_{n}\approx12.1 Hz/s$) is faster than that of passive compensation process ($\nu_{p}\approx5.4 Hz/s$), which can be explained by the fact that the heating generation of resonator is faster than the heating dissipation.

For the tracking system, the parameters of PID control are crucial for total controlled process, the value of key parameters are listed in the table 1. Due to the limitations of equipment and control algorithms, this control time takes about 13s. In fact, if we use the faster control device (such as FPGA) and the better algorithms (such as fuzzy PID control) in subsequent work, this time can theoretically be reduced to the millisecond level. This means that the millisecond-level control time can fully meet the real-time measurement requirements of dynamic low-frequency acceleration (<50Hz), such as earthquakes, submarines, and geological prospecting and other fields.

**Supplementary** **Table S1.** The key parameter of frequency automatic tracking system

| **Control parameter** | **Symbol** | **Value** |
| --- | --- | --- |
| Total control time | $T_{t}$ | 13 |
| Response Velocity | $v_{thermo}$ | 1.792Hz/s |
| Unit response time | $T_{0}$ | 2s |
| Unit voltage compensation | $V_{0}$ | 0.001V |
| Frequency threshold | $f_{th}$ | 1Hz |
| Unit thermo frequency | $f_{thermo}$ | 2.94Hz/μA |
| Over-current protection | $I_{p}$ | 9mA |

**Supplementary Section 3. Performance comparisons for synchronized resonant accelerometer.**


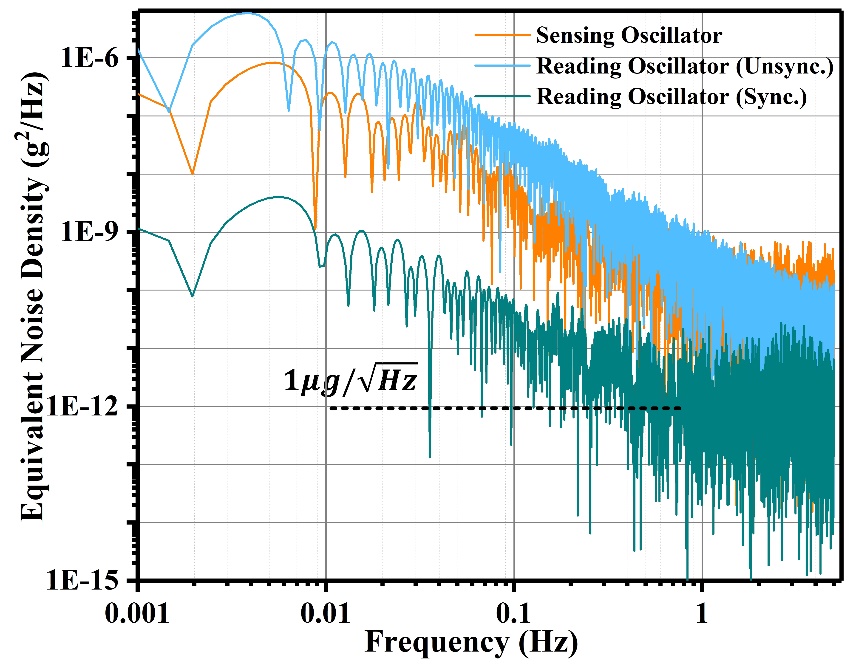


**Supplementary Figure S4.** Power spectral density analysis of synchronized accelerometer. The passive readout oscillator under synchronization state leads to a noise floor of approximately 1μg/$\sqrt{Hz}$ in the frequency range between 1Hz to 5Hz. In comparison, the noise floor of reading oscillator is about 5.31μg/$\sqrt{Hz}$ under non-synchronization state, which was an improvement of 5~6 times. This prove that synchronization has a significant suppression effect on the noise floor of systems.

**Supplementary** **Table S2.** The detailed performance comparisons for synchronized resonant accelerometer.

| **Parameters** | **Sensing oscillator** | **Reading oscillator (Unsync.)** | **Reading oscillator (Sync.)** |
| --- | --- | --- | --- |
| frequency | 128.3kHz | 128.1kHz | 128.3kHz |
| Scale factor | 623Hz/g | N/A | 623Hz/g |
| Resolution | 17.3ug | N/A | 1.91ug |
| Frequency stability | 181.4ppb | 90.8ppb | 19.4ppb |
| Measuring range | ±1g | N/A | ±1g |
| Velocity random walk | 11ug/$\sqrt{s}$ | N/A | 2.05ug/$\sqrt{s}$ |
| Bandwidth | N/A | N/A | 1246Hz |
| Noise floor (@5Hz) | 3.1μg/$\sqrt{Hz}$ | 5.31μg/$\sqrt{Hz}$ | 1μg/$\sqrt{Hz}$ |
